# Insignificant difference in medication adherence to dyslipidemia drugs between visually impaired and non-disabled people in South Korea: A nationwide cohort study using claims records

**DOI:** 10.1371/journal.pone.0307764

**Published:** 2025-01-08

**Authors:** Jong Wook Lee, Hankil Lee, Euna Han, Hye-Young Kang

**Affiliations:** 1 Graduate Program of Industrial Pharmaceutical Science, Yonsei University, Incheon, South Korea; 2 College of Pharmacy, Ajou University, Suwon, Gyeonggi-do, South Korea; 3 College of Pharmacy, Yonsei Institute of Pharmaceutical Sciences, Yonsei University, Incheon, South Korea; National Healthcare Group, SINGAPORE

## Abstract

Incidence of visual impairment (VI) and dyslipidemia is increasing with aging. Although good medication adherence (MA) is a crucial factor in achieving therapeutic goals for dyslipidemia, there is a paucity of studies measuring MA in the visually impaired with dyslipidemia. We investigated whether patients with VI had worse MA to dyslipidemia drugs than non-disabled people and determined the factors affecting MA among patients with VI. Data on dyslipidemia patients with VI were extracted in 2017 from the sample cohort database of the National Health Insurance Service. MA to dyslipidemia drugs was measured for two years based on the proportion of days covered (PDC). Conditional logistic regression analysis was performed to analyze the effect of VI on good MA (PDC ≥0.8). The VI group (0.860) had a larger PDC than the non-disabled group (0.850). The adjusted odds ratio (aOR) for good MA among VI vs. non-disabled individuals was statistically insignificant (1.137, 95% confidence interval:0.958–1.350). Significant factors for poor MA in the VI group were younger age (aOR for 20–39 vs. ≥75 years old: 0.124), lower income (aOR for 9-10th decile (rich) vs. 1-4^th^ decile (poor): 1.771), shorter duration of dyslipidemia (aOR for 1–4 vs. 15 years: 0.416), having lower-level providers sas their main providers (aOR for clinics vs. general/tertiary-care hospitals: 0.545), and having mental diseases (aOR: 0.679). Patients with VI did not have worse MA than non-disabled patients taking dyslipidemia medication.

## Introduction

According to the World Health Organization, at least 2.2 billion people will be visually impaired worldwide by 2022 [1]. Visual impairment (VI) is a disability often caused by aging instead of congenital factors [2,3]. Most patients with VI are older than 50 [1], and the prevalence of VI is expected to increase in the future as the aging population rises.

Compared with non-disabled people, patients with VI experience disability-related barriers to medication administration. Owing to difficulties in identifying medicines and reading medication guides [4], patients with VI mainly take drugs by relying on their memories [5]. As the mobility of patients with VI is restricted without assistance, critical pharmacy visits might be skipped, which may lead to poor medication adherence (MA).

MA is a factor that remarkably affects the treatment outcomes of patients with chronic diseases. In a previous study, higher MA resulted in a lower hospitalization rate and total medical expenditure [6]. Another study revealed that most patients with high adherence had a significantly lower likelihood of hospitalization or emergency room visits than those with low adherence [7].

Dyslipidemia is a common chronic disease that affects two of five Korean adults [8]. If not properly treated, dyslipidemia can lead to cardiovascular diseases, including coronary artery disease, peripheral artery disease, carotid artery stenosis, and heart failure [9]. Visually impaired people are particularly vulnerable to cardiovascular diseases because this cohort includes many elderly patients. The treatment goal for dyslipidemia is to lower the low-density lipoprotein (LDL) cholesterol and triglyceride levels using pharmaceutical therapy, mainly statins. As dyslipidemia is asymptomatic in the early stages, patients may not be aware of the need to take medications and maintain good MA.

Although the prevalence of dyslipidemia among patients with VI is expected to increase due to the high proportion of elderly patients with VI, studies on their MA are lacking. Therefore, in this study, we used population-based big data to assess whether a difference exists in MA to dyslipidemia drugs between patients with and without VI. Furthermore, we investigated whether factors affecting MA to dyslipidemia drugs are distinct among patients with VI compared to non-disabled people to derive effective strategies to improve MA and thus treatment outcomes for those with VI.

## Materials and methods

### Data source

We used the sample cohort database of the National Health Insurance (NHI) Services version 2.2 (NHIS cohort data 2.2, serial number: REQ202201318), a nationally representative dataset comprising a random sample of 2% (approximately 1,000,000) of Korean patients from August 6^th^, 2022 to January 5^th^, 2023. Patient samples were extracted from the claims data of the Korean NHI in 2006 using a stratified random sampling method. These data were the claims records of patient samples from 2002 to 2019 and included limited information on the sociodemographic characteristics of patients, such as age, sex, income, and residence, so that the users could not identify individual participants. Moreover, the data include the diagnosis and procedure codes for NHI-covered inpatient and outpatient services provided to patients, prescription drugs, and selected characteristics of healthcare providers. The study protocol was approved by the Institutional Review Board (IRB No. 7001988-202204-HR-1546-01E). The requirement for informed consent was waived by the board owing to the retrospective nature of the study. All experiments were performed in accordance with the Declaration of Helsinki.

### Study population

From the NHIS sample cohort data, we identified adult patients with dyslipidemia aged 20 or older. Patients with dyslipidemia were defined as follows: 1) have at least two insurance claims records with diagnosis codes of dyslipidemia (International Classification of Diseases, 10th version (ICD-10 code): E78) and prescribed dyslipidemia drugs in 2017 (index year), and 2) have insurance claims records with prescribed dyslipidemia drugs for at least 30 days in 2018–2019 (follow-up period).

In South Korea, VI is defined by the Act on Welfare of Persons with Disabilities [10]. If an individual meets the criteria for VI, they are registered as visually impaired in the national system, and the specific disability type is recorded in the NHIS cohort database. In our study, patients with dyslipidemia were categorized as VI based on their registered disability-type code during the index year. Those without disabilities were classified as non-disabled if they were not registered in the national system. The definition of VI is as follows: 1) A person whose visual acuity in the bad eye (refers to corrected visual acuity) is 0.02 or less; 2) People with visual acuity in the better eye (refers to corrected visual acuity) is 0.2 or less; 3) A person whose field of view in both eyes is less than 10 degrees from the fixation point; 4) A person who has lost more than 1/2 of the visual field in both eyes; 5) People who have double vision within 20 degrees of the central field of view of both eyes.

**Fig 1:**
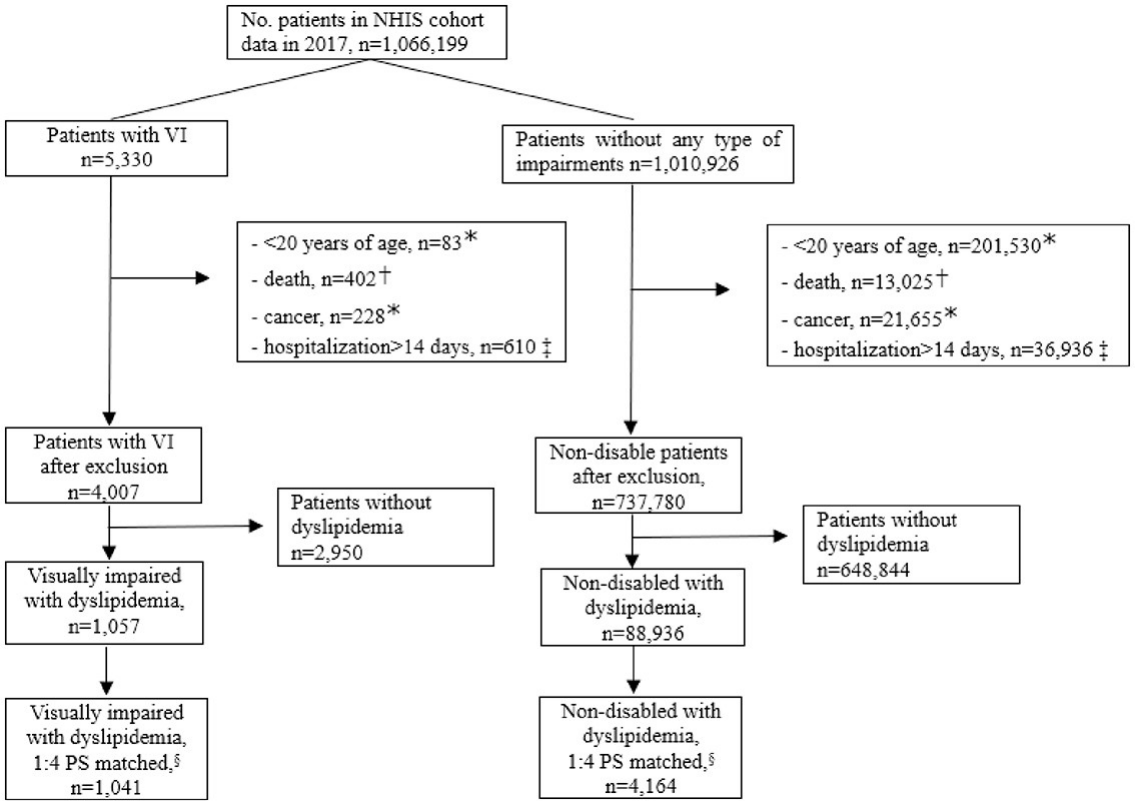
Steps for the selection of the study participants. PS: propensity score, VI: visual impairment. * status during index period, 2017; † status during index and follow-up period, 2017–2019; ‡ status during follow-up period, 2018–2019; § 1:4 PS matched by sex, age, income.

Individuals who died during the follow-up period were excluded. Patients diagnosed with any type of cancer (ICD-10 code C) during the index year were excluded as such diagnosis may affect MA in patients with chronic diseases. Patients hospitalized for more than 14 days during the follow-up period were excluded from the analysis because hospitalization can affect adherence. Non-disabled patients were matched with patients with VI by propensity score (PS) using the variables of age group, income deciles, and Charlson Comorbidity Index (CCI) score (Fig 1) [11]. The distribution of propensity score is presented in S1 Fig.

### Medication adherence measurement

During the 2-year follow-up period, we measured MA by ‘proportion of days covered (PDC),’ calculated by dividing the total number of days supplied with dyslipidemia drugs by the total number of days observed (i.e., 730 days) [12,13]. Some patients were re-prescribed dyslipidemia drugs before the last prescription day. If the prescription was a refill issued by the same prescriber, we assumed that the patient attempted to receive the subsequent prescription in advance. Thus, we defined the last day of the last prescription as the first day of the next prescription to avoid underestimation of the PDC. However, if the prescription was not issued by the same prescriber, we assumed that the patients did not finish taking the medication from the last prescription and switched to a new prescription. Thus, we counted the number of days covered by the drugs from the last prescription to the first day of the new prescription.

Based on the estimated PDC for individuals, we divided the patients into two groups: good (PDC: ≥0.8) and poor MA (PDC: <0.8). A PDC of 0.8 is often used as a standard for evaluating MA in various studies [14–16]. We computed the proportion of good MA among visually impaired and non-disabled dyslipidemia patients, respectively.

**Fig 2:**
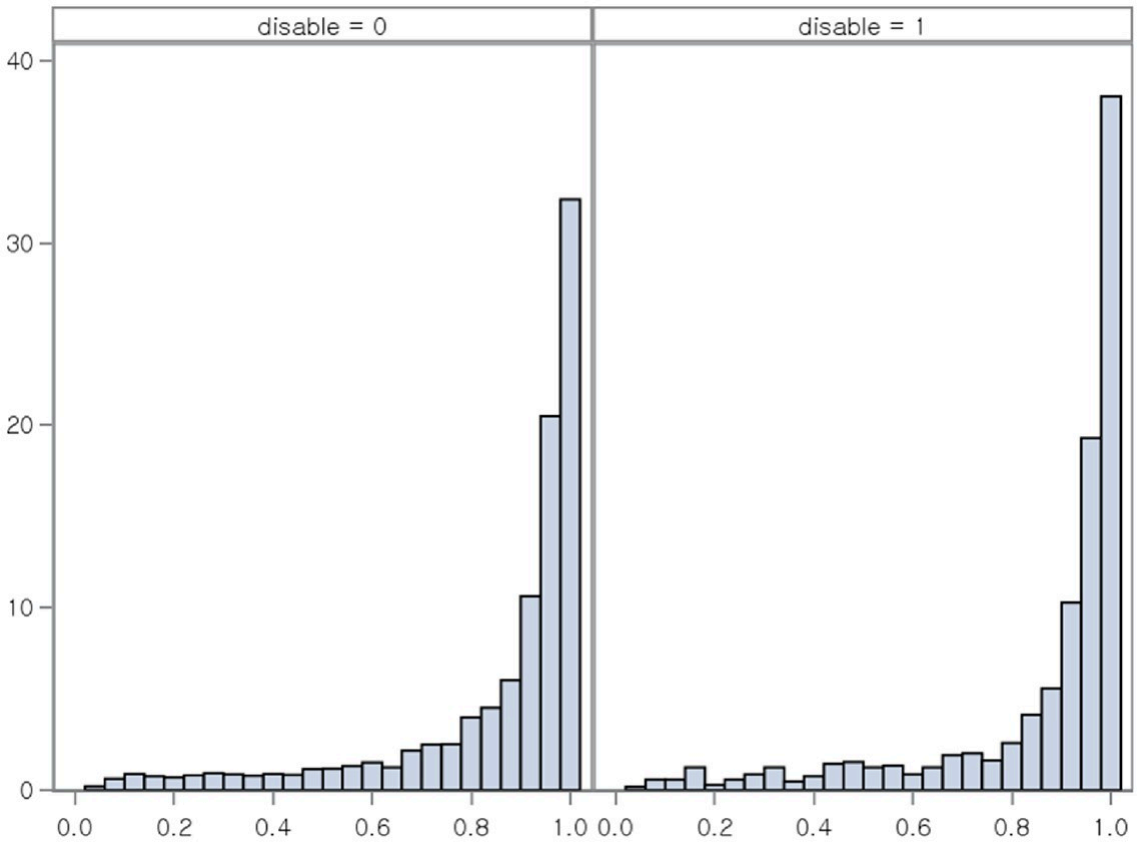
Comparison of concepts for medication adherence measurements: Proportion of days covered and medication persistence. MA: medication adherence. MP: medication persistence. PDC: proportion of days covered.

We measured medication persistence (MP), which highlights whether a patient takes medication continuously without clinically meaningful interruptions. MP is often assessed based on the duration of time a patient takes a medication, from initiation to discontinuation of therapy [17]. We considered patients with poor MP if the interval between the last day of the last prescription and the first day of the next prescription was longer than half of the total prescription days of the last prescription. Otherwise, patients were defined as having good MP. The visual concepts for MA measurements are described in Fig 2.

### Defining dyslipidemia drugs

Oral lipid-lowering drugs for dyslipidemia covered by the Korean NHI include statins (e.g., atorvastatin, fluvastatin, lovastatin, pitavastatin, pravastatin, simvastatin, and rosuvastatin), ezetimibe, fibrates (e.g., bezafibrate, fenofibrate, and gemfibrozil), and omega 3. The primary goal of the pharmacological treatment of dyslipidemia is to lower LDL level [18]. Statins are the most used drugs to lower LDL level. If statins are ineffective at lowering LDL to the target level, even at the maximum doses prescribed, ezetimibe or protein convertase subtilisin/kexin type 9 (PCSK9) inhibitors (e.g., alirocumab or evlocumab) can be co-prescribed with statins. However, PCSK9 inhibitors were not included in our analysis because they became available as NHI-covered drugs in Korea after the follow-up period. If patients with dyslipidemia have high levels of triglycerides, fibrates, or omega 3, single drug therapy or combination therapy with statins is a treatment choice.

### Covariates

To assess the independent association between VI and MA in patients with dyslipidemia, we included various covariates known to affect adherence to drug therapy for chronic diseases. The patients’ sociodemographic characteristics available in the NHIS cohort data were retrieved, including age, sex, income, and residential area (i.e., Seoul (capital city), metropolitan cities, and other areas) in the index year. Income is presented as income deciles, with a higher decile indicating a better economic status.

The baseline health status during the index year was described using the CCI [19], with a higher score indicating a worse health condition. Patients were classified into three groups according to their CCI score: 0, 1, or ≥2. Several comorbid chronic conditions associated with MA were identified in each patient, including hypertension, diabetes mellitus (DM), chronic obstructive pulmonary disease (COPD), and mental diseases [20–23].

We included the types of healthcare institutions (i.e., clinics, hospitals, general hospitals, or tertiary-care hospitals) that were most frequently visited by patients for all causes during the follow-up period as a proxy measure for patients’ health status during the period when MA was assessed.

We examined the severity and duration of dyslipidemia and VI. If patients were prescribed high-dose statins (i.e., atorvastatin 40–80 mg per day or rosuvastatin 20–40 mg per day) or received combination therapy with statins and ezetimibe, they were considered to have severe dyslipidemia [18]. Otherwise, patients were defined as having mild dyslipidemia. Based on the time of the first claim with a diagnosis of dyslipidemia and the prescription of antilipidemic drugs before the index period, we measured the duration of dyslipidemia in each patient. The NHS data provide information on the severity of disability in disabled people. We defined severe VI as a disability level of 1 or 2 and mild VI as a disability level of 3 to 6. The duration of VI was measured from the time of the first claim to the registration of the VI before the index period. As the NHIS cohort data were left-censored until 2002, the maximum duration of dyslipidemia and VI from the index year (2017) was 16 years.

Since we calculated MA using data from 2018–2019, we expect minimal impact from censoring before 2002 on the results, as this time period is significantly distant from 2002. It is highly improbable that disabilities and chronic diseases existing before 2002 would disappear after that period. In our logistic regression analysis, we categorized the duration of VI or dyslipidemia into duration groups. Consequently, the impact of durations exceeding 16 years is considered in the logistic regression results.

### Data analysis

As PDC was not normally distributed in the visually impaired and non-disabled patient groups, we performed a nonparametric Wilcoxon rank-sum test to compare PDC between the two groups [24]. Chi-squared tests were conducted to compare the binary variables of good MA and MP.

Conditional logistic regression analyses were performed to assess whether good MA or MP was associated with VI [25]. To identify whether factors associated with good MA or MP differed depending on the VI status, we performed a separate logistic regression analysis for visually impaired and non-disabled patients.

To examine whether the association between VI and MA/MP differed by age group, duration of VI (acquired or congenital VI), and severity of dyslipidemia, subgroup analyses were performed for the corresponding characteristics. A large proportion of visually impaired individuals belong to the elderly cohort, and a positive association between aging and good MA has been reported in earlier studies [26]. Thus, we assumed that adherence to dyslipidemia drugs among patients with VI might differ between elderly people aged 65 years or older and non-elderly people. Separate conditional logistic regression analyses were performed for each age group.

The extent of difficulty in taking prescribed medications might differ between patients with acquired and congenital VI. Thus, separate logistic regression analyses were performed to compare patients with acquired VI vs. PS-matched non-disabled patients, and patients with congenital or long-term VI vs. PS-matched non-disabled patients.

The NHIS cohort data did not provide information on whether the VI was congenital or acquired. Thus, patients with acquired VI were identified if the diagnosis of VI was observed after 2002 (i.e., left-censoring data point) or claims records were available with diagnosis potentially causing VI from 2002 to 2017, such as uncorrected refractive error (ICD-10 code: H52), cataract (H25, H26, H28.0, H28.1, H28.2), glaucoma (H40-H42), corneal opacity (H17), and diabetic retinopathy (H36) [27]. For patients with VI that do not satisfy any of the two criteria, whether they all had congenial VI was unclear; therefore, these patients were classified as having long-term or congenital VI.

A final subgroup analysis was conducted for the mild and severe dyslipidemia patient groups using separate conditional logistic regression models.

## Results

### Characteristics of the study population

During the index year of 2017, 1,057 adult patients with VI and 88,936 adult patients without any impairment who satisfied the exclusion criteria were identified from the NHIS cohort data 2.2. (Fig 1). The proportion of men in the VI group (57.33%) was significantly higher than that in the non-disabled group (46.59%; p<0.001). The mean (standard deviation, SD) age was 66.35 years (10.93) and 61.77 (11.23) years for the VI and non-disabled groups (p<0.001), respectively. More than 40% of the VI group had an income below the median (1–4 income deciles), whereas 32.61% of the non-disabled group had an income below the median (p<0.001). Approximately 12% of the VI group were eligible to receive Medical Aid, which is four times that of the non-disabled group (3.41%, p<0.001). A relatively large number of VI lived in metropolitan cities, while the proportion of non-disabled people living in capital cities was higher (p = 0.222).

Overall, the VI group had significantly worse health conditions than the non-disabled group, with a higher CCI score (p<0.001); a higher prevalence of comorbid chronic diseases, such as DM (p<0.001), mental diseases (p<0.001), and COPD (p = 0.049); and a higher proportion of patients utilizing general or tertiary care hospitals as their main healthcare providers (19.21 vs. 14.61%, p<0.001). The two groups did not differ in terms of the severity of dyslipidemia (p = 0.103), with approximately one-fifth of patients having dyslipidemia unmanageable by statins. However, the VI group had a longer duration of dyslipidemia (8.01 vs. 7.42 years, P <0.001). Of those with VI, one-fifth had severe VI and approximately two-thirds had experienced VI for more than 10 years. After PS matching based on age, income, and CCI score, significant differences in all characteristics mentioned above disappeared, except for the type of main health care provider (Table 1).

**Table 1:** Basic characteristics of the study participants.

	Before PS matching^1^		After PS matching^1^	
	Patients with VI, n (%)	Non-disabled patients, n (%)	Patients with VI, n (%)	Non-disabled patients, n (%)
Total No. patients	1,057	88,936	1,041	4,164
Sex				
Male	606 (57.33)	41,434 (46.59)*	597 (57.35)	2,380 (57.16)
Female	451 (42.67)	47,502 (53.41)	444 (42.65)	1,784 (42.84)
Age, years (±SD)	66.35±10.93	61.77±11.23*	66.39±10.96	65.74±10.91
20–39	12 (1.14)	2,241 (2.52)	12 (1.15)	49 (1.18)
40–59	263 (24.88)	35,762 (40.21)	259 (24.88)	1,019 (24.47)
60–74	524 (49.57)	38,051 (42.78)	514 (49.38)	2,066 (49.62)
≥ 75	258 (24.41)	12,882 (14.48)	256 (24.59)	1,030 (24.74)
Income deciles^2^				
1–4	449 (42.48)	29,002 (32.61)*	433 (41.59)	1,736 (41.69)
5–8	318 (30.09)	31,865 (35.83)	318 (30.55)	1,268 (30.45)
9–10	290 (27.44)	28,069 (31.56)	290 (27.86)	1,160 (27.86)
CCI score^3^				
0	236 (22.33)	26,771 (30.10)*	231 (22.19)	924 (22.19)
1	356 (33.68)	32,570 (36.62)	352 (33.81)	1,412 (33.91)
≥2	465 (43.99)	29,595 (33.28)	458 (44.00)	1,828 (43.90)
Type of NHS program enrolled				
Medical Aid	128 (12.11)	3,036 (3.41)*	127 (12.20)	505 (12.13)
NHI	929 (87.89)	85,900 (96.59)	914 (87.80)	3,659 (87.87)
Residence				
Seoul (capital city)	209 (19.77))	18,770 (21.11)	209 (20.08)	893 (21.45)
Metropolitan cities	285 (26.96)	22,043 (24.79)	277 (26.61)	1,039 (24.95)
Others	563 (53.26)	48,123 (54.11)	555 (53.31)	2,232 (53.60)
Comorbid conditions				
Hypertension	609 (57.62)	49,854 (56.06)	599 (57.54)	2,428 (58.31)
Diabetes mellitus	462 (43.71)	32,250 (36.26)*	455 (43.71)	1,765 (42.39)
Mental diseases	223 (21.10)	13,433 (15.10)*	220 (21.13)	797 (19.14)
COPD	17 (1.61)	890 (1.00)*	17 (1.63)	72 (1.73)
Severity of dyslipidemia^4^				
Severe	196 (18.54)	17,524 (19.70)	189 (18.16)	809 (19.43)
Mild	861 (81.46)	71,412 (80.30)	852 (81.84)	3,355 (80.57)
Duration of dyslipidemia, years^5^	8.01±4.57	7.42±4.56*	7.99±4.58	7.96±4.61
1–4	301 (28.48)	29,861 (33.58)	299 (28.72)	1,217 (29.23)
5–9	343 (32.45)	28,916 (32.51)	338 (32.47)	1,336 (32.08)
10–14	301 (28.48)	22,492 (25.29)	294 (28.24)	1,151 (27.64)
15–16	112 (10.60)	7,667 (8.62)	110 (10.57)	460 (11.05)
Most-frequently used healthcare institution^6^				
General/Tertiary care hospitals	203 (19.21)	12,996 (14.61)*	202 (19.40)	635 (15.25)*
Hospitals	36 (3.41)	3,214 (3.61)	36 (3.46)	134 (3.22)
Clinics	818 (77.39)	72,726 (81.77)	803 (77.14)	3,395 (81.53)
Severity of VI				
Severe	203 (19.21)	-	200 (19.21)	-
Mild	854 (80.79)	-	841 (80.79)	-
Duration of VI, years^5^	10.66±4.71	-	10.67±4.70	-
1–4	161 (15.23)	-	158 (15.18)	-
5–9	220 (20.81)	-	216 (20.75)	-
10–14	370 (35.00)	-	366 (35.16)	-
15–16	306 (28.95)	-	301 (28.91)	-

CCI: Charlson Comorbidity Index, COPD: chronic obstructive pulmonary disease, NHI: National Health Insurance, NHS: National Health Security, PS: propensity score, SD: standard deviation, VI: visual impairment.

*p<0.05 comparing patients with VI vs non-disabled patients. Unless specified, values for each variable were measured during the index year of 2017, when dyslipidemia patients were identified.

^1^PS matching was conducted using age, sex, income deciles, and CCI score.

^2^The higher the income decile, the better economic status.

^3^Higher CCI score indicates more severe health conditions.

^4^We defined severe dyslipidemia patients as those prescribed high-dose statins (e.g., atorvastatin 40-80mg or rosuvastatin 20-40mg) or co-prescribed statins and ezetimibe.

^5^Becuase the data set is left-censored as of 2002, the maximum duration from the index year of 2017 is 16 years.

^6^It was based on the most frequently visited health care institutions for all causes during the follow-up period of 2018 to 2019.

### Impact of visual impairment on medication adherence

**Fig 3:**
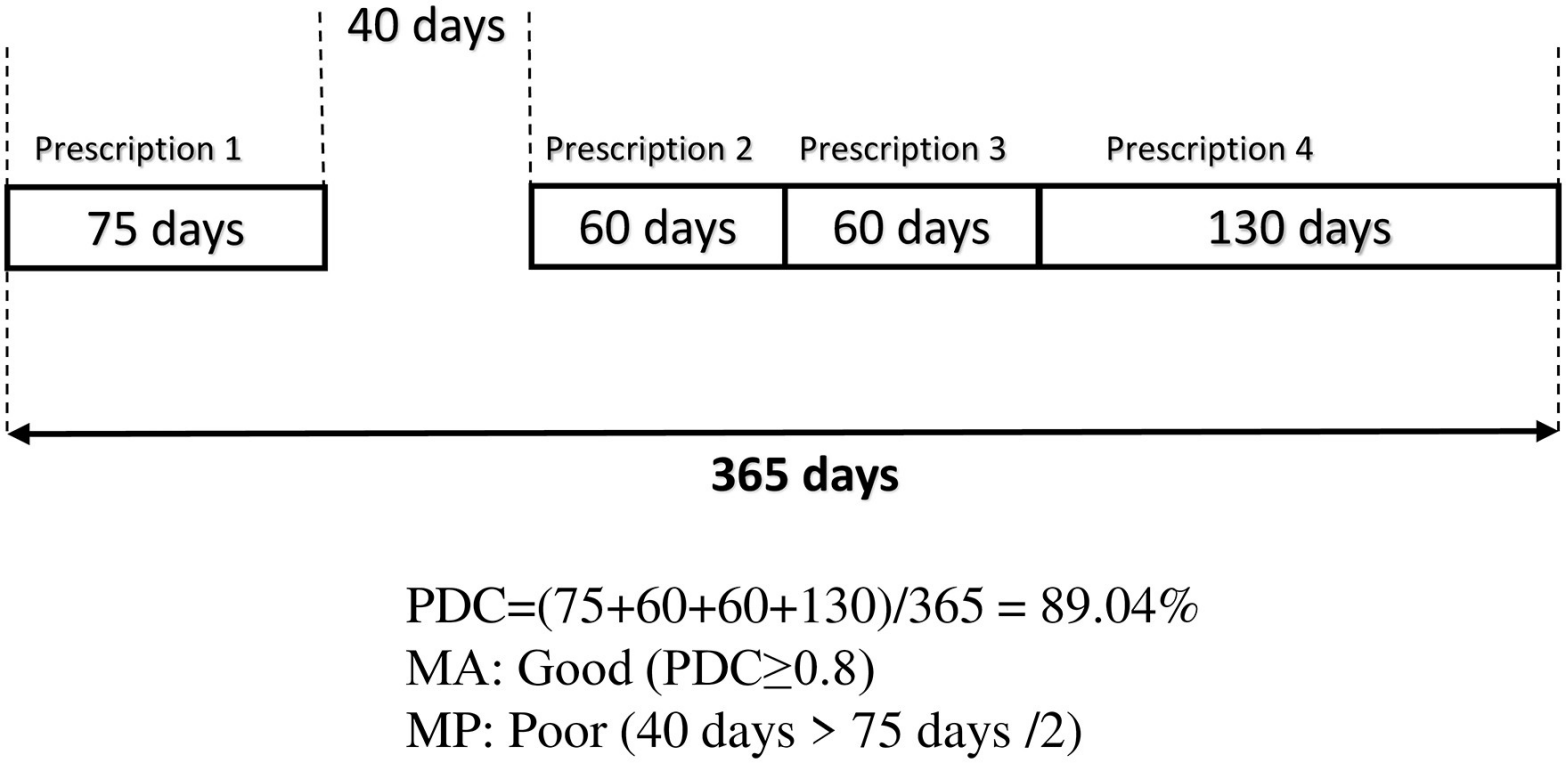
Distribution of medication adherence measured by proportion of days covered among patients with dyslipidemia (2018–2019). (Left: Non-disabled patients, Right: Visually impaired patients).

The 2-year average PDC for dyslipidemia drugs among patients with VI was 0.860, suggesting that, on average, these patients were prescribed dyslipidemia drugs for approximately 314 days per year (365 days × 0.860) (Table 2). The 2-year mean PDC for non-disabled patients was 0.850, which was significantly lower than that for patients with VI (p<0.05). The proportion of patients with good MA (78.77% vs. 76.30%, p = 0.091) or the rate of MP (61.96% vs. 59.49%, p = 0.145) did not significantly differ between patients with and without disabilities. Fig 3 shows the distribution of PDC, which was not normally distributed in either group. The PDC at or near 1.0 was > 30% in both groups, indicating a trend toward higher adherence. After adjusting for covariates in the multiple conditional logistic regression analysis, VI was found to be a non-significant factor influencing MA (aOR, 1.137; 95% CI, 0.958–1.350) and MP (aOR, 1.086; 95% CI, 0.941–1.254) among patients with dyslipidemia in South Korea (Table 3).

**Table 2:** Comparison of medication adherence between visually impaired and non-disabled patients with dyslipidemia.

	Visually impaired (n = 1,041)			Non-disabled (n = 4,164)	p-value	
	Total (n = 1,041)	Severe (n = 200)	Mild (n = 841)		p^1^	p^2^
Mean PDC (SD)	0.860 (0.215)	0.869 (0.211)	0.858 (0.216)	0.850 (0.216)	0.004	0.268
Patients with good medication adherence, ^3^%	78.77	79.50	78.60	76.30	0.091	0.779
Rate of persistence, %	61.96	64.00	61.47	59.49	0.145	0.508

PDC: proportion of days covered, SD: standard deviation.

^1^comparing visually impaired patients vs. non-disabled patients.

^2^comparing severe vs. mild visual impairment.

^3^Defined as patients with ≥ 0.8 of PDC.

**Table 3:** Conditional logistic regression analysis results for the association between visual impairment and medication adherence among patients with dyslipidemia in South Korea.

	Good medication *adherence* aOR [95% CI]	Good medication *persistence* aOR [95% CI]
Visual impairment		
Non-disabled	Ref.	Ref.
Visually impaired	1.137 [0.958–1.350]	1.086 [0.941–1.254]
Residence		
Seoul (capital city)	Ref.	Ref.
Metropolitan cities	0.862 [0.691–1.076]	0.991 [0.820–1.197]
Others	0.962 [0.793–1.169]	1.042 [0.884–1.228]
Most-frequently used healthcare institution^1^		
General/Tertiary care hospitals	Ref.	Ref.
Hospitals	0.678 [0.427–1.077]	0.656 [0.446–0.964]
Clinics	0.498[0.396–0.627]	0.550 [0.457–0.661]
Comorbid conditions^2^		
Hypertension	1.426 [1.219–1.667]	1.277 [1.117–1.459]
Diabetes mellitus	1.655 [1.366–2.006]	1.231 [1.047–1.448]
Mental diseases	0.816 [0.668–0.996]	0.807 [0.682–0.954]
COPD	0.992 [0.550–1.789]	0.862 [0.526–1.410]
Severity of dyslipidemia^3^		
Severe	0.907 [0.749–1.098]	0.890 [0.756–1.048]
Mild	Ref.	Ref.
Duration of dyslipidemia, years^4^		
1–4	0.643 [0.483–0.855]	0.687 [0.543–0.870]
5–9	0.840 [0.634–1.114]	0.856 [0.681–1.076]
10–14	1.019 [0.762–1.363]	0.942 [0.747–1.188]
15–16	Ref.	Ref.

aOR: adjusted odds ratio. CI: confidence interval. ref: reference group.

Non-disabled patients were propensity-score matched with visually impaired patients by age, sex, income deciles, and Charlson Comorbidity Index score. Unless specified, values for each variable were measured during the index year of 2017, when dyslipidemia patients were identified.

^1^It was based on the most frequently visited health care institutions for all causes during the follow-up period of 2018 to 2019.

^2^For each comorbid condition, those without the condition were defined as reference group.

^3^We defined severe dyslipidemia patients as those prescribed high-dose statins (e.g., atorvastatin 40-80mg, rosuvastatin 20-40mg) or co-prescribed statins and ezetimibe.

^4^Becuase the data set is left-censored as of 2002, the maximum duration from the index year of 2017 is 16 years.

### Factors affecting medication adherence

To identify whether the factors affecting MA or MP differed depending on the VI status, we performed a separate logistic regression analysis for visually impaired and non-disabled patients (Table 4). Overall, the factors affecting MA or MP for dyslipidemia drugs were similar, regardless of whether patients had VI. In fact, common factors that are significantly associated with poor MA or MP in both the VI and non-disabled groups include younger age (aOR for MA of patients with VI aged 20–39 years vs. ≥75 years: 0.124, 95% CI: 0.033–0.466; and aOR for MA of non-disabled patients: 0.176, 95% CI: 0.095–0.325), shorter duration since diagnosis of dyslipidemia (aOR for MA of patients with VI having dyslipidemia for 1–4 years vs. ≥15 years: 0.416, 95% CI: 0.216–0.803; and aOR for MA of non-disabled patients: 0.654, 95% CI: 0.495–0.865), and having lower-level health care institutions as their main health care providers (aOR for MA of patients with VI having clinics as main providers vs. general or tertiary-care hospitals: 0.545, 95% CI: 0.347–0.858; and aOR for MA of non-disabled patients: 0.520, 95% CI: 0.412–0.657). Although having comorbid chronic diseases, such as hypertension and DM, in addition to dyslipidemia led to better MA or MP for both groups, comorbid mental diseases caused the opposite (aOR for MP of patients with VI: 0.679, 95% CI: 0.487–0.948; and aOR for MP of non-disabled patients: 0.827, 95% CI: 0.700–0.977).

**Table 4:** Logistic regression analysis results for factors associated with medication adherence for patients with dyslipidemia: Separate analysis for visually impaired and non-disabled patients.

	Visually impaired patients (n = 1,041)		Non-disabled patients (n = 4,164)	
	Good medication *adherence* aOR [95% CI]	Good medication *persistence* aOR [95% CI]	Good medication *adherence* aOR [95% CI]	Good medication *persistence* aOR [95% CI]
Sex				
Male	Ref.	Ref.	Ref.	Ref.
Female	1.138 [0.816–1.587]	0.902 [0.682–1.192]	0.965 [0.825–1.127]	0.907 [0.793–1.036]
Age, years				
20–39	0.124 [0.033–0.466]	0.136 [0.034–0.544]	0.176 [0.095–0.325]	0.173 [0.087–0.341]
40–59	0.622 [0.382–1.015]	0.444 [0.294–0.672]	0.425 [0.336–0.538]	0.548 [0.450–0.669]
60–74	0.864 [0.562–1.327]	0.767 [0.542–1.084]	0.717 [0.584–0.881]	0.794 [0.673–0.936]
≥ 75	Ref.	Ref.	Ref.	Ref.
Income deciles^1^				
1–4	Ref.	Ref.	Ref.	Ref.
5–8	1.242 [0.845–1.827]	1.315 [0.937–1.844]	1.103 [0.916–1.329]	0.989 [0.840–1.164]
9–10	1.771 [1.150–2.727]	1.296 [0.909–1.846]	1.140 [0.931–1.396]	1.080 [0.909–1.285]
CCI score^2^				
0	Ref.	Ref.	Ref.	Ref.
1	1.051 [0.689–1.602]	1.139 [0.785–1.654]	1.125 [0.919–1.377]	1.046 [0.874–1.253]
≥2	1.212 [0.748–1.965]	1.172 [0.770–1.783]	1.185 [0.942–1.491]	1.190 [0.973–1.456]
Type of NHS program				
Medical Aid	Ref.	Ref.	Ref.	Ref.
NHI	0.669 [0.383–1.170]	0.664 [0.416–1.061]	0.686 [0.527–0.893]	0.843 [0.676–1.051]
Residence				
Seoul (capital city)	Ref.	Ref.	Ref.	Ref.
Metropolitan cities	0.763 [0.472–1.234]	1.029 [0.696–1.523]	0.901 [0.726–1.119]	0.977 [0.810–1.177]
Others	0.739 [0.479–1.139]	0.964 [0.682–1.364]	1.016 [0.839–1.229]	1.034 [0.879–1.217]
Comorbid conditions^3^				
Hypertension	1.207 [0.874–1.667]	1.082 [0.824–1.422]	1.523 [1.309–1.772]	1.390 [1.220–1.583]
Diabetes mellitus	1.511 [1.025–2.228]	1.222 [0.882–1.691]	1.436 [1.193–1.727]	1.125 [0.962–1.315]
Mental diseases	0.887 [0.593–1.328]	0.679 [0.487–0.948]	0.881 [0.724–1.073]	0.827 [0.700–0.977]
COPD	0.939 [0.254–3.472]	0.689 [0.249–1.92]	0.834 [0.462–1.508]	0.929 [0.565–1.527]
Severity of dyslipidemia^4^				
Severe	0.748 [0.504–1.109]	0.875 [0.619–1.236]	0.909 [0.755–1.095]	0.827 [0.756–1.041]
Mild	Ref.	Ref.	Ref.	Ref.
Duration of dyslipidemia, years^5^				
1–4	0.416 [0.216–0.803]	0.431 [0.260–0.716]	0.654 [0.495–0.865]	0.746 [0.592–0.941]
5–9	0.582 [0.302–1.121]	0.668 [0.405–1.101]	0.868 [0.657–1.146]	0.901 [0.718–1.130]
10–14	0.715 [0.363–1.406]	0.720 [0.432–1.201]	1.029 [0.773–1.369]	0.966 [0.768–1.216]
≥15	Ref.	Ref.	Ref.	Ref.
Most-frequently used healthcare institution^6^				
General/Tertiary care hospitals	Ref.	Ref.	Ref.	Ref.
Hospitals	1.075 [0.371–3.114]	0.645 [0.294–1.416]	0.537 [0.342–0.843]	0.650 [0.439–0.961]
Clinics	0.545 [0.347–0.858]	0.504 [0.350–0.726]	0.520 [0.412–0.657]	0.580 [0.481–0.700]
Severity of VI				
Severe	1.000 [0.662–1.512]	1.055 [0.745–1.493]	-	-
Mild	Ref.	Ref.	-	-
Duration of VI, years^5^				
1–4	1.005 [0.607–1.664]	0.921 [0.604–1.404]	-	-
5–9	1.118 [0.694–1.801]	1.287 [0.868–1.910]	-	-
10–14	0.815 [0.555–1.197]	0.894 [0.643–1.242]	-	-
15–16	Ref.	Ref.	-	-

aOR: adjusted odds ratio. CI: confidence interval. CCI: Charlson Comorbidity Index, COPD: chronic obstructive pulmonary disease, NHI: National Health Insurance, NHS: National Health Security.

Unless specified, values for each variable were measured during the index year of 2017, when dyslipidemia patients were identified.

^1^The higher the income decile, the better economic status.

^2^Higher CCI score indicates more severe health conditions.

^3^ For each comorbid condition, those without the condition were defined as reference group.

^4^We defined severe dyslipidemia patients as those prescribed high-dose statins (e.g., atorvastatin 40-80mg or rosuvastatin 20-40mg) or co-prescribed statins and ezetimibe.

^5^Becuase the data set is left-censored as of 2002, the maximum duration from the index year of 2017 is 16 years.

^6^It was based on the most frequently visited health care institutions for all causes during the follow-up period of 2018 to 2019.

Income level was the only variable that had a significant impact on MA for those with VI but did not significantly impact MA for non-disabled patients. Compared to the lowest income group (1–4 deciles), those from the highest income group (9–10 deciles) had a significantly higher chance of good MA among patients with VI (aOR: 1.771, 95% CI: 1.150–2.727).

### Results from subgroup analyses

To investigate whether the impact of VI on MA or MP varies depending on certain characteristics of visually impaired patients, we conducted subgroup analyses for selected characteristics, including age group (elderly or non-elderly), duration of VI (acquired or congenital VI), and severity of dyslipidemia (severe or mild). No significant effect of VI on MA or MP for dyslipidemia drugs was observed in any of the subgroups (Table 5).

**Table 5:** Subgroup analysis results for the association between visual impairment and medication adherence among patients with dyslipidemia in South Korea.

	Adjusted odds ratio for good medication adherence											
	Age group, years				Duration of VI				Severity of dyslipidemia			
	≥65 (n = 2,916)		<65 (n = 2,289)		Acquired VI (n = 760) vs. non-disabled (n = 3,040)		Congenital or long-term (≥17 years) VI (n = 281) vs. non-disabled (n = 724)		Severe (n = 998)		Mild (n = 4,207)	
	MA	MP	MA	MP	MA	MP	MA	MP	MA	MP	MA	MP
Visual impairment												
Non-disabled	Ref.	Ref.	Ref.	Ref.	Ref.	Ref.	Ref.	Ref.	Ref.	Ref.	Ref.	Ref.
Visually impaired	1.146	1.090	1.294	1.100	1.140	1.049	1.147	1.201	1.281	1.055	1.156	1.126
Residence												
Seoul (capital city)	Ref.	Ref.	Ref.	Ref.	Ref.	Ref.	Ref.	Ref.	Ref.	Ref.	Ref.	Ref.
Metropolitan cities	0.876	1.033	0.894	0.956	0.836	1.033	0.938	0.911	0.551	1.266	0.816	0.887
Others	1.043	1.114	0.824	0.909	0.881	1.003	1.171	1.134	0.642	0.859	0.924	1.052
Most-frequently used healthcare institution^1^												
General/Tertiary care hospitals	Ref.	Ref.	Ref.	Ref.	Ref.	Ref.	Ref.	Ref.	Ref.	Ref.	Ref.	Ref.
Hospitals	1.023	0.680	0.687	0.721	0.805	0.656	0.478	0.689	0.155	0.375	0.821	0.845
Clinics	0.656*	0.622*	0.413*	0.453*	0.537 *	0.549 *	0.396*	0.549*	0.190*	0.480*	0.566*	0.590*
Comorbid conditions												
Hypertension	1.227	1.074	1.646*	1.570*	1.387*	1.239*	1.462*	1.345*	1.421	0.990	1.381*	1.238*
Diabetes mellitus	1.526*	1.124	1.969*	1.515*	1.858*	1.280*	1.249	1.105	1.493	1.155	1.717*	1.196
Mental diseases	0.714*	0.818	1.005	0.782	0.732*	0.750*	1.162	1.029	0.368*	0.523*	0.798	0.819*
COPD	1.190	0.823	0.792	1.400	0.966	0.802	1.307	1.095	0.902	0.329	0.952	1.061
Severity of dyslipidemia^2^												
Severe	0.810	0.831	0.950	0.925	0.820	0.774*	1.181*	1.265	-	-	-	-
Mild	Ref.	Ref.	Ref.	Ref.	Ref.	Ref.	Ref.	Ref.	-	-	-	-
Duration of dyslipidemia, years^3^												
1–4	0.509*	0.745	0.798	0.690	0.592*	0.713*	0.897	0.633	0.966]	0.775	0.619*	0.724*
5–9	0.761	0.865	0.910	0.901	0.741	0.868	1.290	0.826	1.375	0.881	0.828	0.931
10–14	0.888	0.986	1.194	1.050	0.967	0.944	1.343	0.940	2.057	0.806]	0.977	1.052
15–16	Ref.	Ref.	Ref.	Ref.	Ref.	Ref.	Ref.	Ref.	Ref.	Ref.	Ref.	Ref.

MA: good medication adherence. MP: good medication persistence. VI: visual impairment.

*95% confidence interval does not include 1.0.

For each subgroup analysis, non-disabled patients were propensity-score matched with visually impaired patients by age, sex, income deciles, and Charlson Comorbidity Index score. Unless specified, values for each variable were measured during the index year of 2017, when dyslipidemia patients were identified.

^1^It was based on the most frequently visited health care institutions for all causes during the follow-up period of 2018 to 2019.

^2^We defined severe dyslipidemia patients as those prescribed high-dose statins (e.g., atorvastatin 40-80mg, rosuvastatin 20-40mg) or co-prescribed statins and ezetimibe.

^3^Becuase the data set is left-censored as of 2002, the maximum duration from the index year of 2017 is 16 years.

## Discussion

Contrary to our projection, patients with VI did not have worse MA or MP than non-disabled patients taking dyslipidemia medications in South Korea. Subgroup analyses by age, VI duration, and dyslipidemia severity repeatedly confirmed the insignificant effect of VI on MA or MP for dyslipidemia drugs.

To the best of our knowledge, no previous study assessed MA to dyslipidemia medications among patients with VI. However, several studies have been published on MA in other chronic diseases, such as hypertension and DM, and in patients with various types of disabilities. In a Korean study conducted in 2017, in which MA to antihypertensive drugs was analyzed for people with various disabilities using claims data, as performed in our study, MA was lower in the disabled group than in the non-disabled group; however, the difference after adjusting for confounding variables was less than 1%, indicating clinically insignificant results [28]. Cho et al. investigated MA to hypertension medications using Korean Medical Panel data, which measure MA based on responses of panel participants. Based on their findings, disability did not significantly affect MA [29]. A study conducted in 2008 using claims records reported that MA to antihypertensive drugs was significantly lower in patients with VI than in non-disabled people [30]. These studies provide inconsistent results regarding the effect of disabilities on MA, potentially due to the type of data (i.e., health insurance claims records or questionnaire survey) used, methods used to measure MA, and recentness of the study conducted.

We investigated whether socioeconomic and clinical characteristics differentially affect MA or MP in visually impaired and non-disabled patients with dyslipidemia. Notably, the factors associated with MA did not significantly differ between the two groups, except for income. Although non-disabled patients did not show significantly different MA or MP according to income status, visually impaired patients in the highest income classes (9–10 deciles) had an approximately 1.8 times higher MA than those in the lowest income classes (1–4 deciles). As Korea has a universal health security system, financial barriers to outpatient services and prescription drugs are limited. Most dyslipidemia drugs are covered by the National Health Insurance; thus, patients are required to copay only 20–30% of the total healthcare expenditures for medication therapy. Moreover, patients in the lowest economic class are enrolled in the Public Assistant Medical Aid program, which does not require patient copayment for covered services. Regardless of this system, our results revealed that a patient with VI living in poverty had a significantly lower chance of maintaining good MA than a patient with VI living in affluence.

The significant impact of income levels on MA in visually impaired individuals compared to its lack of significance in the non-disabled, may stem from various reasons. Lower-income individuals with visual impairment face multiple barriers to healthcare access, including challenges with transportation, costs associated with healthcare facilities, limited availability of services, and insufficient support for medication management. Additionally, they have restricted access to education and health information, leading to lower health literacy levels that affect their ability to comprehend and adhere to medication instructions. Furthermore, the financial stress associated with visual impairment and lower income can contribute to mental health issues, further influencing medication adherence. Limited resources may also hinder visually impaired individuals from accessing or affording assistive technologies or caregivers that could support medication adherence.

Of note, the proportion of study participants with income below the median (1–4 income deciles) was higher in the VI group (42.48%) than in the non-disabled group (32.61%, Table 1), and the proportion of Medical Aid beneficiaries in the VI group (12.11%) was four times higher than that in the non-disabled group (3.41%, Table 1). This finding suggests the need for special attention to financially vulnerable patients with VI to minimize obstacles to maintaining good MA. Addressing these socioeconomic factors, such as improving healthcare access, providing education, offering financial assistance, and promoting social support, can significantly contribute to enhancing MA among lower-income individuals with visual impairment. VI.

The patient characteristics commonly influencing MA or MP in both visually impaired and non-disabled patients with dyslipidemia were age, comorbid DM, comorbid mental disease, duration of dyslipidemia, and type of healthcare institution. In both groups, older patients had better MA and MP, showing a linear relationship between aging and good MA or MP. Previous studies reported the same phenomenon. Colantonio et al. measured compliance with statin therapy in American adults between 2007 and 2014. The relative ratio for adherence was 0.71 (95% CI:0.70–0.72) in the 21–44-year-old group compared to the 55–64-year-old group and 0.86 (95% CI:0.85–0.87) in the 45–54-year-old group, suggesting that MA was lower in the younger group [31]. Awareness of the increased risk of CV due to the non-use of statins may be low among young people, which may result in poor MA.

Regardless of the presence of VI, patients with dyslipidemia who most frequently visited clinics among the various types of healthcare institutions for all-cause medical treatments tended to have lower MA than those who most often visited general or tertiary care hospitals (aOR: 0.504–0.580). We used this variable as a proxy measure for the study participants’ overall health status, assuming that patients were sicker if they received healthcare services predominantly from higher-level healthcare institutions. Thus, the study results can be interpreted as healthier study participants (i.e., those with clinics as their major healthcare providers) tended to have poor MA, which might be due to less concerns about their health relative to sicker study participants. Considering the unique practice patterns of South Korea, different interpretations are possible. Although most clinics in South Korea operate on a walk-in basis, hospitals operate on appointment systems. Therefore, if patients with dyslipidemia receive healthcare mainly from hospitals, general hospitals, or tertiary care hospitals, they may visit hospitals and refill their prescriptions according to their scheduled appointments. Consequently, the MA measured using the PDC for these patients can be high.

In both the VI and non-disabled groups, MA was low when the duration of dyslipidemia was short. This result is probably because patients with newly diagnosed dyslipidemia are relatively young and are not aware of the risk of developing CVD due to poor management of dyslipidemia. In addition, as dyslipidemia itself does not exhibit symptoms, it is difficult for newly diagnosed patients to consider dyslipidemia as a potentially serious condition. According to the results of Yilmaz, who estimated the medication possession ratio for patients with Crohn’s, the younger the age and shorter the duration of the disease, the lower the MA [32].

This study had several limitations, most of which are associated with insurance claims data characteristics. First, we assessed patients’ MA to dyslipidemia drugs using the PDC, which can be measured as high if claims records show that patients regularly refill prescriptions. However, we could not determine whether the patient actually took the medication; this is a common limitation in MA studies that use claims data. Therefore, MA measured by the PDC based on claims records may be overestimated. Second, as the data source used to measure MA was healthcare utilization data, patients in blind spots of medical use were excluded from the analysis. Thus, untreated patients with physical or economic difficulties accessing medical care were excluded from the data. In this study, the VI group had a higher proportion of low-income and older patients than the non-disabled group (Table 1). Accordingly, the actual MA of the VI group may be lower than that revealed in our study. Third, our study measured the PDC and MP only for NHI-covered drug therapy. We did not have information on patients’ medication behavior for NHI-uncovered drug therapies. Fourth, the statistical analysis may be biased due to the small number of patients aged 20–39. Dyslipidemia, being a chronic condition primarily affecting the elderly, resulted in a small proportion of individuals under 40 years old. To mitigate age-related effects, we matched the age distribution between the two groups; however, there is a potential for sparse data bias in our study. Finally, we did not include provider or drug regimen characteristics as covariates in the regression model used to assess the impact of VI on MA or MP. For the characteristics of healthcare providers, the claims data used for this study comprise information on the type and location of healthcare institutions and the medical subject of prescribers. As the patient’s drug-taking behavior was not considered to be influenced by either the location of healthcare institutions or the medical subject of prescribers, we did not include these variables as covariates. Complex drug regimens and frequent dosing often impede patients’ ability to follow scheduled medications. In addition, drug side effects can lead patients terminating medication use. However, statin treatment, which is the predominant drug therapy for dyslipidemia, is usually prescribed as a single dose per day, and its side effects are rare. Thus, we did not include these variables as covariates in our analysis. Although statin combination with other drugs for the treatment of dyslipidemia is infrequent, we incorporated this into our analysis as a variable of severe dyslipidemia, which was defined if patients were prescribed high-dose statins or received combination therapy with statins and ezetimibe.

In summary, based on the PDC and MP measured from claims data, we confirmed that VI did not hamper the maintenance of good MA and persistence among patients with VI under NHI-covered medication in South Korea. Unlike non-disabled patients, financial difficulty significantly affected patients with VI after scheduled medication therapy for dyslipidemia. Thus, special attention to financially vulnerable VI patients is necessary to improve the management of dyslipidemia.

## Supporting information

S1 Fig Distribution of propensity score. (JPG)

## References

[1] World Health Organization. Blindness and vision impairment. WHO [Internet]. 2022 Oct [cited 2023 Feb 7]. https://www.who.int/news-room/fact-sheets/detail/blindness-and-visual-impairment

[2] Klaver CC, Wolfs RC, Vingerling JR, Hofman A, de Jong PT. Age-specific prevalence and causes of blindness and visual impairment in an older population: the Rotterdam Study. Archives of ophthalmology. 1998;116(5):653–8. doi: 10.1001/archopht.116.5.653 9596502

[3] Bowl MR, Dawson SJ. Age-related hearing loss. Cold Spring Harbor perspectives in medicine. 2019;9(8):a033217. doi: 10.1101/cshperspect.a033217 30291149 PMC6671929

[4] Killick K, Macaden L, Smith A, Kroll T, Stoddart K, Watson MC. A scoping review of the pharmaceutical care needs of people with sensory loss. Int J Pharm Pract. 2018 Oct;26(5):380–386. doi: 10.1111/ijpp.12456 29920822

[5] Riewpaiboon A. How the blind cope with problems of medicine utilization: a study—58—in Bangkok, Thailand. Pharmacoepidemiol Drug Saf. 2009 Aug;18(8):708–12. 19455569 10.1002/pds.1771

[6] Sokol MC, McGuigan KA, Verbrugge RR, Epstein RS. Impact of medication adherence on hospitalization risk and healthcare cost. Med Care. 2005;43(6):521–530. doi: 10.1097/01.mlr.0000163641.86870.af 15908846

[7] Gillespie CW, Morin PE, Tucker JM, Purvis L. Medication Adherence, Health Care Utilization, and Spending Among Privately Insured Adults With Chronic Conditions in the United States, 2010–2016. Am J Med. 2020;133(6):690–704.e19. doi: 10.1016/j.amjmed.2019.12.021 31987798

[8] The Korean Society of Lipid and Atherosclerosis. Dyslipidemia Fact Sheets in Korea, 2022. KSoLA. 2022.

[9] Gupta M, Tummala R, Ghosh RK, Blumenthal C, Philip K, Bandyopadhyay D, et al. An update on pharmacotherapies in diabetic dyslipidemia. Prog Cardiovasc Dis. 2019;62(4):334–341. doi: 10.1016/j.pcad.2019.07.006 31442512

[10] Minister of Government Legislation. Korean Law Information Center. https://www.law.go.kr/LSW/eng/engMain.do?eventGubun=060124.

[11] Brookhart MA, Schneeweiss S, Rothman KJ, Glynn RJ, Avorn J, Stürmer T. Variable selection for propensity score models. Am J Epidemiol. 2006;163(12):1149–1156. doi: 10.1093/aje/kwj149 16624967 PMC1513192

[12] Karve S, Cleves MA, Helm M, Hudson TJ, West DS, Martin BC. Prospective validation of eight different adherence measures for use with administrative claims data among patients with schizophrenia. Value Health. 2009;12(6):989–995. doi: 10.1111/j.1524-4733.2009.00543.x 19402852

[13] Raebel MA, Schmittdiel J, Karter AJ, Konieczny JL, Steiner JF. Standardizing terminology and definitions of medication adherence and persistence in research employing electronic databases. Med Care. 2013;51(8 Suppl 3):S11–S21. doi: 10.1097/MLR.0b013e31829b1d2a 23774515 PMC3727405

[14] Chapman RH, Benner JS, Petrilla AA, Tierce JC, Collins SR, Battleman DS, et al. Predictors of adherence with antihypertensive and lipid-lowering therapy. Arch Intern Med. 2005 May 23;165(10):1147–52. doi: 10.1001/archinte.165.10.1147 . 15911728

[15] Ozaki AF, Choi AS, Le QT, Ko DT, Han JK, Park SS, et al. Real-World Adherence and Persistence to Direct Oral Anticoagulants in Patients With Atrial Fibrillation: A Systematic Review and Meta-Analysis. Circ Cardiovasc Qual Outcomes. 2020 Mar;13(3):e005969. Epub 2020 Mar 9.doi: 10.1161/CIRCOUTCOMES.119.005969 . 32148102

[16] Ho PM, Rumsfeld JS, Masoudi FA, McClure DL, Plomondon ME, Steiner JF, et al. Effect of medication nonadherence on hospitalization and mortality among patients with diabetes mellitus. Arch Intern Med. 2006;166(17):1836–1841. doi: 10.1001/archinte.166.17.1836 17000939

[17] Sikka R, Xia F, Aubert RE. Estimating medication persistency using administrative claims data. Am J Manag Care. 2005;11(7):449–457. 16044982

[18] Authors/Task Force Members; ESC Committee for Practice Guidelines (CPG); ESC National Cardiac Societies. 2019 ESC/EAS guidelines for the management of dyslipidaemias: Lipid modification to reduce cardiovascular risk. Atherosclerosis. 2019 Nov;290:140–205. Epub 2019 Aug 31. Erratum in: Atherosclerosis. 2020 Jan;292:160–162. Erratum in: Atherosclerosis. 2020 Feb;294:80–82. doi: 10.1016/j.atherosclerosis.2019.08.014 . 31870624

[19] Sundararajan V, Henderson T, Perry C, Muggivan A, Quan H, Ghali WA. New ICD-10 version of the Charlson comorbidity index predicted in-hospital mortality. J Clin Epidemiol. 2004 Dec;57(12):1288–94. doi: 10.1016/j.jclinepi.2004.03.012 . 15617955

[20] Rublee DA, Chen SY, Mardekian J, Wu N, Rao P, Boulanger L. Evaluation of cardiovascular morbidity associated with adherence to atorvastatin therapy. Am J Ther. 2012;19(1):24–32. doi: 10.1097/MJT.0b013e3181ee707e 20838204

[21] Aarnio E, Martikainen J, Winn AN, Huupponen R, Vahtera J, Korhonen MJ. Socioeconomic Inequalities in Statin Adherence Under Universal Coverage: Does Sex Matter?. Circ Cardiovasc Qual Outcomes. 2016;9(6):704–713. doi: 10.1161/CIRCOUTCOMES.116.002728 27756795

[22] El-Saifi N, Moyle W, Jones C, Tuffaha H. Medication Adherence in Older Patients With Dementia: A Systematic Literature Review. J Pharm Pract. 2018;31(3):322–334. doi: 10.1177/0897190017710524 28539102

[23] Lopes J, Santos P. Determinants of Non-Adherence to the Medications for Dyslipidemia: A Systematic Review. Patient Prefer Adherence. 2021;15:1853–1871. Published 2021 Aug 24. doi: 10.2147/PPA.S319604 34465984 PMC8403077

[24] Dutta S, Datta S. A rank-sum test for clustered data when the number of subjects in a group within a cluster is informative. Biometrics. 2016;72(2):432–440. doi: 10.1111/biom.12447 26575695 PMC4870168

[25] Sperandei S. Understanding logistic regression analysis. Biochem Med (Zagreb). 2014;24(1):12–18. Published 2014 Feb 15. doi: 10.11613/BM.2014.003 24627710 PMC3936971

[26] Cohen MJ, Shaykevich S, Cawthon C, Kripalani S, Paasche-Orlow MK, Schnipper JL. Predictors of medication adherence postdischarge: the impact of patient age, insurance status, and prior adherence. J Hosp Med. 2012;7(6):470–475. doi: 10.1002/jhm.1940 22473754 PMC3575732

[27] Pascolini D, Mariotti SP. Global estimates of visual impairment: 2010. Br J Ophthalmol. 2012;96(5):614–618. doi: 10.1136/bjophthalmol-2011-300539 22133988

[28] Kim SJ, Choi HC, Lee CM, Oh S-W, Joh H-K, Oh B, et al. Severity of Disability an Antihypertensive Medication Adherence in Korea. Korean J Fam Pract. 2017;7(6):926–932.

[29] Cho SJ, Kim J. Factors associated with nonadherence to antihypertensive medication. Nurs Health Sci. 2014;16(4):461–467. doi: 10.1111/nhs.12145 24823924

[30] Park JH, Park JH, Lee SY, Kim SY, Shin Y, Kim SY. Disparities in antihypertensive medication adherence in persons with disabilities and without disabilities: results of a Korean population-based study. Arch Phys Med Rehabil. 2008;89(8):1460–1467. doi: 10.1016/j.apmr.2007.12.045 18674981

[31] Colantonio LD, Rosenson RS, Deng L, Monda KL, Dai Y, Farkouh ME, et al. Adherence to Statin Therapy Among US Adults—60—Between 2007 and 2014. J Am Heart Assoc. 2019 Jan 8;8(1):e010376. doi: 10.1161/JAHA.118.010376 . 30616455 PMC6405715

[32] Yilmaz H. Young age and shorter duration of Crohn’s disease are associated with non-adherence to taking medication. North Clin Istanb. 2022 Feb 10;9(1):8–13. doi: 10.14744/nci.2021.08634 . 35340313 PMC8889204

