# Supplementary figures and images for: Insignificant difference in medication adherence to dyslipidemia drugs between visually impaired and non-disabled people in South Korea: A nationwide cohort study using claims records
Source: PLoS One. 2025 Jan 8;20(1):e0307764. doi: 10.1371/journal.pone.0307764 (PMC11709254; doi:10.1371/journal.pone.0307764)

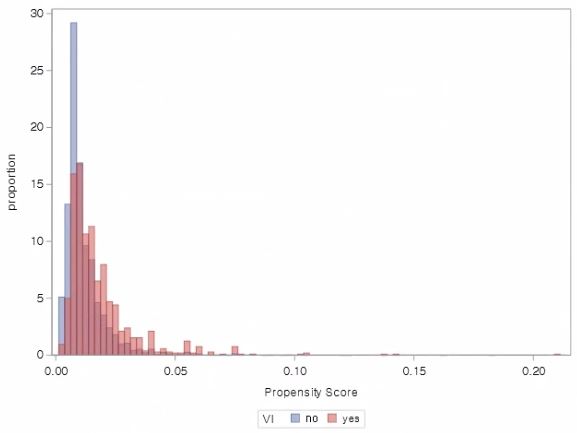

Supplement: S1 Fig — (JPG) [file pone.0307764.s001.JPG]
